# Comparison of a Low-Cost Miniature Inertial Sensor Module and a Fiber-Optic Gyroscope for Clinical Balance and Gait Assessments

**DOI:** 10.1155/2019/9816961

**Published:** 2019-09-25

**Authors:** Daniel Roetenberg, Claudia Höller, Kevin Mattmüller, Markus Degen, John H. Allum

**Affiliations:** ^1^Hocoma AG, Volketswil, Switzerland; ^2^FHNW University of Applied Sciences and Arts Northwestern Switzerland, School of Life Sciences, Muttenz, Switzerland; ^3^Department of ORL, University of Basel Hospital, Basel, Switzerland

## Abstract

**Objective:**

To investigate whether a microelectromechanical system (MEMS) inertial sensor module is as accurate as fiber-optic gyroscopes when classifying subjects as normal for clinical stance and gait balance tasks.

**Methods:**

Data of ten healthy subjects were recorded simultaneously with a fiber-optic gyroscope (FOG) system of SwayStar™ and a MEMS sensor system incorporated in the Valedo® system. Data from a sequence of clinical balance tasks with different angle and angular velocity ranges were assessed. Paired *t*-tests were performed to determine significant differences between measurement systems. Cohen's kappa test was used to determine the classification of normal balance control between the two sensor systems when comparing the results to a reference database recorded with the FOG system. Potential cross-talk errors in roll and pitch angles when neglecting yaw axis rotations were evaluated by comparing 2D FOG and 3D MEMS recordings.

**Results:**

Statistically significant (*α*=0.05) differences were found in some balance tasks, for example, “walking eight tandem steps” and various angular measures (*p* < 0.03). However, these differences were within a few percent (<2.7%) of the reference values. Tasks with high dynamic velocity ranges showed significant differences (*p*=0.002) between 2D FOG and 3D MEMS roll angles but no difference between 2D FOG and 2D MEMS roll angles. An almost perfect agreement could be obtained for both 2D FOG and 2D MEMS (*κ*=0.97) and 2D FOG and 3D MEMS measures (*κ*=0.87) when comparing measurements of all subjects and tasks.

**Conclusion:**

MEMS motion sensors can be used for assessing balance during clinical stance and gait tasks. MEMS provides measurements comparable to values obtained with a highly accurate FOG. When assessing pitch and roll trunk sway measures without accounting for the effect of yaw, it is recommended to use angle and angular velocity measures for stance, and only angular velocity measures for gait because roll and pitch velocity measurements are not influenced by yaw rotations, and angle errors are low for stance.

## 1. Introduction

Technological advances and clinical research have shown that body-worn sensors measuring angular velocity (gyroscopes) and/or the acceleration of the trunk can accurately quantify balance during stance and gait tasks [1, 2], enabling detection of potential fallers [3] and discrimination between clinically different balance disorders [4].

The sensors used for these purposes must be accurate over different ranges of angular velocity, low velocity ranges (<0.5°/s) for stance tests on a firm surface [4], and high velocity ranges (>100°/s) for more dynamic tasks such as rising from a stool. To detect possible deviations of body sway compared to normal reference ranges when standing with eyes open or closed on a firm surface, tests typically used clinically, highly accurate, low-noise, and low-drift sensors are required [5]. In contrast, when performing a comparison of body dynamics to those of healthy subjects, observed when rising from a stool and then walking forward, a sensor with a large working range and high resolution is required [6]. Tasks with intermediate ranges of sway amplitudes, such as those of normal walking, require a mix of these requirements in order to identify elderly “fallers” [7]. The question is whether technologic improvements in microelectromechanical system (MEMS) motion sensors forming the basis of low-cost (approximately 10 times cheaper) and lightweight inertial measurement units (IMUs) are able to replace relatively more expensive, heavier, but more accurate (drift 10 times less) fiber-optic gyroscopes (FOGs) used for assessing a wide spectrum of balance tasks [2, 4].

**Figure 1:**
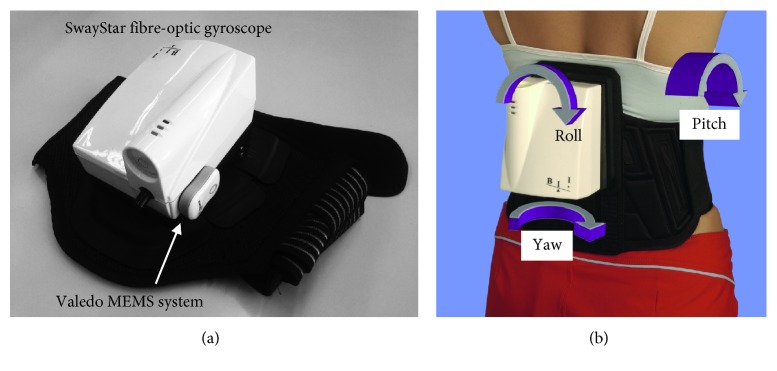
(a) SwayStar (FOG) mounted on a converted motorcycle belt with a Valedo (MEMS) sensor attached to its side. (b) The SwayStar system mounted on a subject. The SwayStar motion measurement axes (pitch and roll) are, as shown, sensor-based.

A further method to reduce the costs of balance measuring devices is to use a sensor system measuring only roll and pitch motion thereby ignoring yaw motion (Figure 1(b)) under the assumption that motion about the yaw axis has a negligible influence on roll and pitch measures for most clinical stance and gait tests, except those involving turning. As this approach is often used with FOG systems, a confirmation of the negligible influence of yaw would permit interchange of reference value databases [5] collected with both devices.

In this study, we investigated whether a 2D or 3D MEMS motion sensor could be used as a cheaper lightweight alternative to measuring balance control in the form of angular sway velocity at the lower trunk with accurate FOGs. As small sensors can be placed easily at other locations on the body, an affirmative result would pave the way for the use of such sensors in different body locations and provide the basis for a comprehensive body-mounted motion analysis system. Our primary hypothesis was that a MEMS system would provide a comparable level of accuracy (kappa > 0.8) in classifying normal balance test results as a FOG system. We did not compare the MEMS motion to optical motion capture system because, unlike the 2 systems we compared in this study, motion capture systems are not portable and not quick to start, requiring the attachment of several optical “markers.”

## 2. Methods

### 2.1. Measurement Systems

A fiber-optic gyroscope system SwayStar, manufactured by Balance International Innovations GmbH (Switzerland), was used as this is supplied with an extensive healthy control reference database of several clinical stance and gait balance tasks for subjects in the age range of 6 to 80 years [5]. This system measures the angular velocities of the trunk in sensor coordinates near the center of mass (around L3–L5) by means of two orthogonally placed fiber-optic gyroscopes (FOG). These record trunk sway velocities in sensor coordinates in the pitch and roll directions (Figure 1(b)). Rotations about the yaw axis are not measured with this system. Data were sampled at 100 Hz and sent unfiltered via a wireless Bluetooth connection to the PC, where the data were low-pass-filtered at 30 Hz. Angular deviations in the roll and pitch direction were calculated using trapezoid integration of angular velocity recordings from the sensors after any velocity spikes (due to a communication error) in the data were removed with a 3-sample filter examining differences between angular accelerations over the 3 samples and then low-pass-filtering with a low-pass finite impulse response filter with a cutoff of 30 Hz. Spikes were removed by examining if the neighboring accelerations were of different sign and exceeded the mean plus 1.5 standard deviations of all accelerations in the recording, and then the sample with a spike was replaced with a linearly extrapolation value from the neighboring samples. The sensors have a specified drift of 6°/hr, a noise level (random walk) of 0.04°/s per √hr, and a maximum range of ±256°/s sampled with a resolution of 16 bits at 100 Hz. The dimensions of the sensor box mounted on a converted motorcycle belt are 15 cm × 11 cm × 9 cm, and the weight with the sensors is approximately 750 grams.

For the microelectromechanical system (MEMS), one sensor system from the Valedo® products, developed and manufactured by Hocoma AG (Switzerland), was used. The standard application of these sensors is to measure pelvic and spinal movements in order to assess movement parameters and to provide training as part of a physiotherapy plan [8]. The dimensions of each sensor are 4.2 cm × 3.2 cm × 1.6 cm, and the weight is 18 grams. The sensor module consists of a 3D MEMS gyroscope, 3D accelerometer, and 3D magnetometer, together with an onboard microprocessor, battery, and Bluetooth Low Energy (BLE) module. The gyroscope has a typical drift of 30°/hr and a noise level of 0.02°/s per √Hz, and samples have a resolution of 14 bits at 1000 Hz. The internal microprocessor runs an extended Kalman filter fusing the data of all three sensing elements outputting drift-free orientation [9]. The data of the magnetometers were not taken into account in the Kalman filter sensor fusion to eliminate any effect of magnetic disturbances [10]. Data from the sensor were transferred to the client (PC) by means of the BLE Notify operation at a rate of 50 Hz. Sampled data consisted of the orientation of the sensor module in quaternion format with respect to an earth-fixed reference system.

To obtain 3D angular velocity, the received quaternion samples were differentiated with respect to time [11]. Differentiation of the quaternion reduces the effect of gyroscope offset fluctuations and drift in comparison with the directly measured gyroscope signals because the orientation output is corrected by the sensor fusion scheme. The disadvantage is that noise in the orientation samples can cause spikes in the angular velocity derivatives. Therefore, a Hampel filter was applied using the MATLAB (MathWorks) application. This removed spikes by replacing each sample with the median of six surrounding samples [12]. Because the implemented Bluetooth protocol did not ensure that all data packages were received, occasional missing data were linearly interpolated. After the interpolation stage, the data were filtered by means of a second-order low-pass Butterworth filter with a cutoff frequency at the Nyquist frequency of 25 Hz.

The lower trunk angles measured with the MEMS system were calculated using two methods. The first technique involved applying the 3D Tilt/Twist extraction based on the orientation of the sensor [13]. The second was based on the time integration of the roll and pitch angular velocities, yielding 2D sensor-based angles, as used by the SwayStar system. When rotations around the vertical axis (yaw) are not taken into account, these will result in cross-talk between the roll and pitch angles because rotations are not commutative. The effect of this cross-talk was investigated by analyzing the differences between the 2D and 3D angle calculation methods of the 3D MEMs with the 2D angle calculation of the FOG sensor system. Cross-talk does not occur for the angular velocity measures as these are local derivatives.

From the two sets of sampled sensor data, the following measures were extracted for analysis: peak-to-peak range (difference between maximum and minimum value during the task) and 90% range (difference between 95% and 5% percentile values when the peak-to-peak range of sampled values was divided into 40 bins and a histogram of the task recording samples built after assigning samples to these bins), for both angular velocities and angles in the pitch (sagittal plane) and roll (lateral plane) direction. Therefore, the data extraction yielded the following 8 measures:

- Peak-to-peak range, roll, angle
- 90% range, roll, angle
- Peak-to-peak range, pitch, angle
- 90% range, pitch, angle
- Peak-to-peak range, roll, angular velocity
- 90% range, roll, angular velocity
- Peak-to-peak range, pitch, angular velocity
- 90% range, pitch, angular velocity

### 2.2. Experimental Procedures

During the clinical stance and gait tasks, a Valedo (MEMS) sensor was held on the side of the SwayStar (FOG) sensor as shown in Figure 1(a) using double-sided adhesive tape. The mechanical alignment between Valedo and SwayStar coordinate systems was determined using an optimization algorithm as described by Chardonnes et al. [14]. We considered this a better clinical comparison of the devices than mounting both devices to a gyro test-table. Time synchronization between the recordings of the two measurement systems was performed by finding the delay of maximum cross-correlation between the two angular velocity signals of both systems for each trial and correcting sample times for this delay.

Data of 10 young healthy subjects (8 male, 2 female, age: 19–34 years) were recorded with the FOG and MEMS sensor systems simultaneously. We planned to compare between Valedo and SwayStar sensor measurements for 10 subjects and then if several trends for differences were observed to expand the data set to 20 subjects. As described below, the results showed either statistically significant differences or no differences, with a few trends. Therefore, an expansion of the data set was not considered necessary. The 9 tasks evaluated with both sensor systems are listed below in the order these were performed, that is, in the same order as for the reference database [5]. These tasks are considered to represent the full dynamic range of clinically relevant balance assessments [2, 5]. All standing tasks had a predefined duration of 20 seconds. The recording of the walking tasks was ended when the subject completed the task, for example, reached 3 meters or walked 8 tandem steps:

- Standing on two legs with eyes open, on a normal (firm) surface
- Standing on two legs with eyes closed, on a foam surface
- Standing on one leg with eyes closed, on a normal surface
- Walking 8 tandem steps with eyes open
- Getting up from a stool and walking 3 meters
- Walking 3 meters while pitching the head up and down
- Walking 3 meters with eyes closed
- Walking up and down a set of stairs (2 steps up and 2 down)
- Walking 8 meters with eyes open

If subjects were not able to complete a task (due to loss of balance which mostly occurred for the “standing on one leg, eyes closed,” task for which the mean duration for healthy young subjects is 12 sec [5]), the task was not repeated; however, the data were removed from the analysis. The total time required to record the tasks was approximately 10 minutes per subject. The study was approved by the local ethical committee responsible for the University of Basel Hospital (approval EKNZ 2015-071).

## 3. Analysis

For the data comparison, the differences between the FOG and MEMS measurements were expressed as absolute values as well as percentage values. The 8 extracted measures from both sensor systems were compared with reference data from 88 age- and gender-matched healthy subjects recorded by Hegeman et al. [5] (using SwayStar). Pearson's correlation coefficient *r* was calculated to evaluate the correlation of the peak-to-peak measures between the two sensor systems (task specific as well as for all tasks). Paired *t*-tests were performed to determine whether there was a significant difference between means obtained from the 2 devices for the same task measure. For this comparison, all eight measures of all 10 subjects and 9 tasks were normalized relative to the mean value of the normal reference database to account for the differences in magnitudes between tasks: for example, between the differences in the magnitudes of pitch velocity for the task of standing on 2 legs with eyes open and the task of getting up off a stool. Quoted *p* values in the results are before any Bonferroni correction for multiple corrections. The comparisons were made across all tasks and for each task separately. Data from the FOG and MEMS were also compared to the clinically relevant 95% limit of the reference database, looking for values less or greater than this limit. Cohen's kappa test was performed in order to assess the interrater classification accuracy (the number of measures classified as within and outside the normal reference range for the Valedo system compared to the SwayStar system) between the two sensor systems. As both sensor systems measure the movements and outcome variables independently, we therefore considered the systems as independent raters.

## 4. Results

Results of 3 of the 9 tasks performed are presented here in detail. These 3 tasks cover the range from low body dynamics (represented by “standing on two legs with eyes open”) to high dynamics (“get up and go, and then walk 3 meters”). All graphs and tables present both the 2D MEMS and 3D MEMS data. In the sections “2D data processing” and “2D vs. 3D data processing,” the comparison between the 2D and 3D angle calculations is described in further detail for all tasks.

### 4.1. Stance Task: Standing on Two Legs with Eyes Open

**Figure 2:**
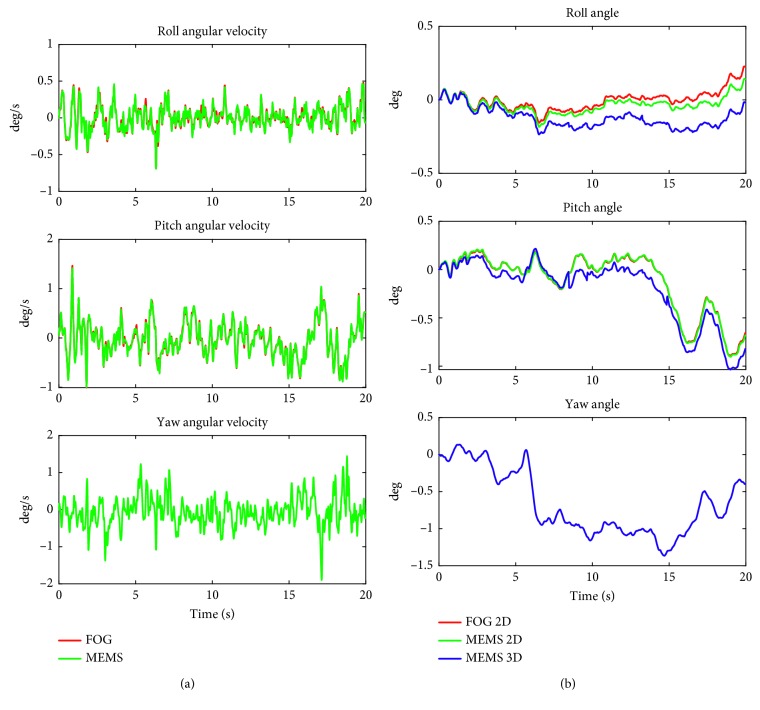
Angular velocity data (a) and angles (b) in the lateral/roll plane (upper), sagittal/pitch plane (middle), and the axial/yaw plane (lower) of the FOG and MEMS sensors for standing on two legs with eyes open on a normal surface task. The red lines depict the 2D FOG data, the green lines the 2D MEMS, and the blue lines the 3D MEMS angle calculations. Note that the velocity traces overlay. The pitch angle 2D traces for FOG and MEMS 2D also overlay. For the yaw angular velocity and yaw angle, only the MEMS data are available.

Figure 2 shows the angular velocity and angle traces for a typical recording for the task “standing on two legs with eyes open on a normal surface.”

In Figure 2, the difference in angles between the two 2D recordings at the end of the 20 seconds recording is less than 0.1 degree. The Pearson correlation coefficient *r* between the 2D MEMS and 2D FOG values of the peak-to-peak values of all subjects is higher than 0.98 for both the angular velocity and angle signals in the pitch and roll planes. The 3D MEMS roll angle has a correlation coefficient of 0.851 with the roll angle of the FOG; the corresponding pitch angle correlation is 0.968. Angular velocity results in both roll and pitch are highly correlated with *r* > 0.99.

Table 1 compares the reference values of the matching age group [5] with the results for FOG and MEMS systems as well as the mean differences between the systems for recordings of all subjects performing the task “standing on two legs with eyes open.” The FOG versus 2D MEMS and FOG versus 3D MEMS sensor values are listed as absolute and relative values (the error between both systems as a percentage of the mean reference data). The *p* value of the paired *t*-test is listed in the table. It can be observed that only roll angle (90% range) and pitch angular velocity (90% range) data are significantly different between the FOG and 2D MEMS measures, whereas the corresponding peak-to-peak values do not show any significant differences. Furthermore, with a Bonferroni correction for multiple comparisons, only pitch angular velocity (90% range) remains significant. There were no significant differences between FOG and 3D MEMS angle values (Table 1).

Note that the differences in 3D are only presented in Table 1 for the angle values; the pitch and roll angular velocities are equal for 2D and 3D. For yaw angles and angular velocities, no FOG reference values are available.

For the other stance tasks, the following was observed: “Standing on two legs with eyes closed, on a foam surface” showed significant differences between FOG and 2D MEMS for both roll and pitch angular velocities (roll: *p*=0.002; pitch: *p* < 0.001, MEMS lower values), as well as the angle in roll plane (*p*=0.02). The task “standing on one leg with eyes closed, on a normal surface” showed no significant differences.

### 4.2. Gait Tasks: Get Up and Go 3 Meters

**Figure 3:**
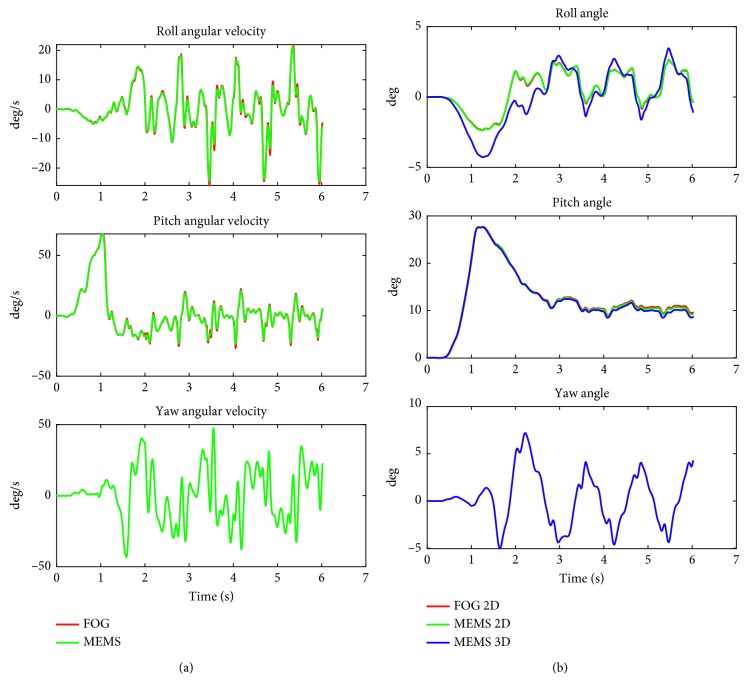
Angular velocity data (a) and angles (b) in the roll plane (upper) and pitch plane (middle) and yaw plane (lower) of the FOG and MEMS sensors for the “get up and go 3 meters” task. The red lines illustrate the 2D FOG data, the green lines the 2D MEMS, and the blue lines the 3D MEMS angle calculations. Note that the velocity traces overlay. The 2D FOG and 2D MEMS roll traces overlay. The pitch angle 2D traces for FOG and MEMS also overlay with the 3D MEMS traces. For the yaw angular velocity and yaw angle, only the MEMS data are available.

Figure 3 shows a typical recording of the angular velocity and angle traces for “get up and go 3 meters” task (a dynamic gait task). Similar to the stance task shown in Figure 2, the biggest deviation can be observed in the 3D MEMS roll angle. The subject rotated axially when getting up and during walking. This yaw rotation is not recorded with the 2D FOG and causes a different projection in the roll plane when compared with the 3D MEMS angles. Across the test population, this difference is significant (Table 2). The MEMS angular velocities and 2D roll and pitch angles of all subjects have a very high correlation (>0.97) with the FOG data. The 3D MEMS roll angle has a correlation of 0.911 with the FOG data, and for the 3D pitch angle, the corresponding correlation is 0.999.

Table 2 shows the reference values of the matching age group in comparison with the FOG and 2D MEMS and 3D MEMS for recordings of all subjects. The relative error between the 2D FOG and the 2D MEMS compared to the mean reference values is 5.73% for the 90% roll angle range but this would not be significant after Bonferroni correction. The difference between the 2D FOG and 3D MEMS roll angle measures is, however, much larger, 18.7%, and more significant (*p*=0.002) (Table 2). Thus, the roll angle is underestimated by the 2D systems. Peak-to-peak pitch velocity was underestimated by the MEMS system.

For the other gait tasks listed below, the differences between the 2D FOG and 3D MEMS roll angles were not significant:

- Walking 3 meters while pitching the head up and down (not significant (ns) with *p*=0.187)
- Walking 3 meters with eyes closed (ns, *p*=0.945)
- Walking up and down a set of stairs (ns, *p*=0.824)
- Walking 8 meters with eyes open (ns, *p*=0.469)

For the pitch angles, no significant differences were observed.

### 4.3. Semi-Gait Task: Walking 8 Tandem Steps with Eyes Open

**Figure 4:**
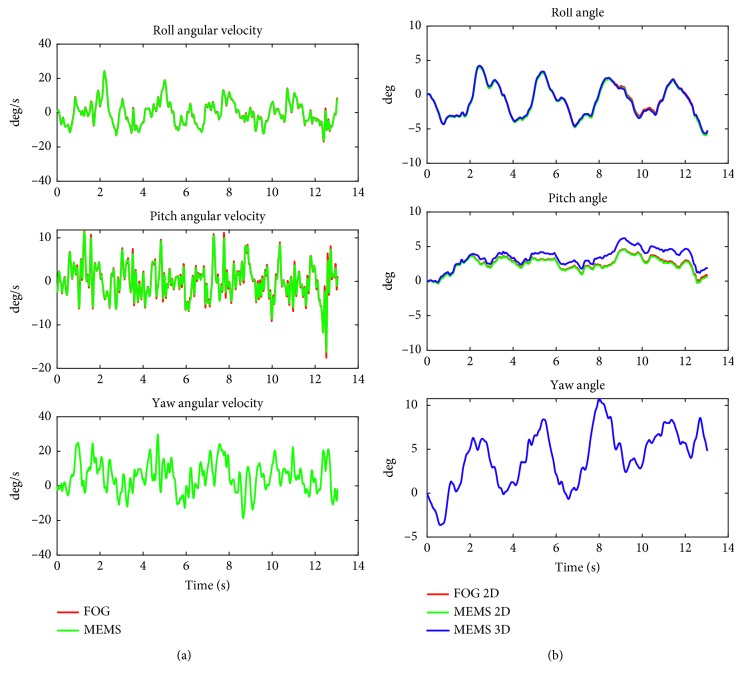
Angular velocity data (a) and angles (b) in the roll plane (upper) and pitch plane (middle) and yaw plane (lower) of the FOG and MEMS sensors for walking 8 tandem steps with eyes open task. The red lines represent the 2D FOG, the green lines the 2D MEMS, and the blue lines the 3D MEMS angle calculations. Note that roll angle traces overlay as do pitch angle FOG and MEMS 2D traces. All velocity traces overlay. The yaw angle is only available for MEMS 3D.

Figure 4 shows angular velocity and angle traces of a typical recording for “walking 8 tandem steps with eyes open.” This is a classical clinical task with body motion alternating between tandem stance and gait. The FOG and 2D MEMS data show very high correlations (*r* > 0.99 for both pitch and roll angle and angular velocity); FOG and 3D MEMS data all show *r* > 0.93.

Table 3 shows the reference values with the comparison of the FOG and MEMS for the task “Walking 8 tandem steps with eyes open” for all recordings. It can be seen that there were no significant differences for pitch and roll angles. However, the 90% angular velocities in both planes differ with respect to those of the MEMS system, which underestimated these measures.

### 4.4. Classification Accuracy between the Sensor Systems

#### 4.4.1. 2D Data Processing

For all tasks, except trials were subjects lost their balance control, the eight extracted measures for both the 2D FOG and 2D MEMS were checked for lying within or outside the normal reference range defined by the 95% limits of the reference database. If the data are within the reference 95% range, clinically, the recording would be considered normal [5].

Because of the loss of balance, six recordings were not taken into account in the analyses (6 *∗* 8=48 variables). Four of these records were due to subjects losing their balance prior to task completion (20 secs) for the task, “Standing on one leg with eyes closed.” The lower 5% limit of duration for this task is 14.7 secs [5].

Table 4 presents the resulting contingencies. Based on these values, Cohen's kappa was calculated and yielded a result of *κ*=0.969. This is usually interpreted as an almost perfect agreement [15].

The single measurement that was inside the range as measured by the MEMS but outside with the FOG was a peak-to-peak value of the angular velocity (no differences were detected for the corresponding 90% range values because single peaks or outliers are filtered out when calculating the 90% range value.). Note that as we compared with 95% reference range values, some values outside the normal range are to be expected.

#### 4.4.2. 2D vs. 3D Data Processing

The 3D angles and angular velocity measures measured with the MEMS and 2D FOG measures were compared with the reference database and the FOG similar to the 2D MEMS comparisons presented in the previous paragraph. For stance tasks that have low ranges, the differences between the 2D and 3D calculations were in the same range as the noise level of the MEMS sensors because the tasks involved limited axial rotation. Thus, divergences in comparison with the reference database were not expected. In contrast, in some recordings of the “get up and go 3 meters” and walking tasks, axial rotation caused a significant “cross-talk” between roll and pitch angles that resulted in a slightly higher number (6) of false-negatives when comparing the angles with the normal reference values. Nonetheless, the Kappa value is 0.868, which is also considered as an almost perfect agreement [15].

**Figure 5:**
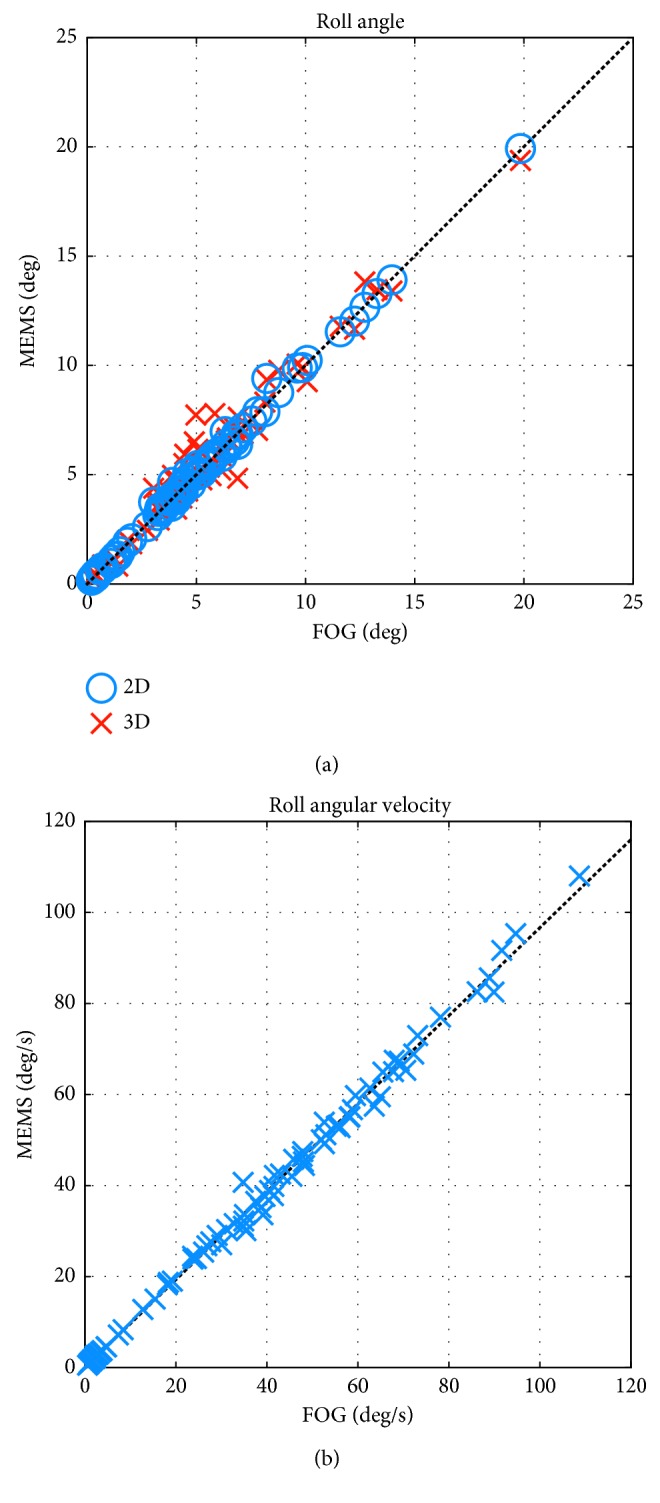
(a) Regression of peak-to-peak roll angle FOG vs MEMS 2D (blue circles) and MEMS 3D (red crosses). (b) Peak-to-peak roll angular velocity FOG vs MEMS.

In Figure 5(a), the regressions between the 2D FOG and 2D MEMS, and 2D FOG and 3D MEMS peak-to-peak roll angles are plotted. In Figure 5(b), 2D FOG and MEMS roll angular velocities (peak-to-peak) are plotted. Data are for all subjects and recordings. The correlation coefficient *r* value for the 2D MEMS angle is 0.991. The 3D MEMS angles have an *r* value of 0.922. The angular velocity *r* value is 0.994. All results are highly significant (*p* < 0.001). Similar regression results could be observed for pitch angle and pitch angular velocities (*r* > 0.98). The classification matrixes described in Tables 4 and 5, and the regressions of Figure 5 indicate that the differences between the two measurement systems are small from a clinical viewpoint across all tasks, including those not described in detail above. Otherwise, for example, the regressions of Figure 5 would be less significant.

## 5. Discussion

In this study, we have tested whether low-cost MEMS motion sensors can provide comparable accuracy as highly accurate fiber-optic gyroscopes to assess balance tasks, which require low noise, minimum drift, and a high resolution across the range of angular sway and sway velocity induced by the balance tasks. We could also assess whether cross-talk errors on pitch and roll angular measures due to not recording yaw angular velocity are significant. If comparable in accuracy and with insignificant cross-talk errors, then MEMS motion sensors can be used to compare extracted balance measures with reference values obtained with highly accurate fiber-optic gyroscopes recording pitch and roll angular velocities. Our main findings were, firstly, that except for the get up and go test, there were no significant differences between 2D FOG and 3D MEMS roll and pitch angle measures. Secondly, angular velocities were slightly underestimated with the MEMS system. Thus, the analyses of the 2D MEMS data showed almost perfect agreement with the FOG data with an interrater classification accuracy of *κ*=0.969 when comparing the measures with those of a normal reference data set [5]. In summary, although as described above, for some tasks and some measures statically significant differences were found, further analysis showed that all these differences were within a few percent of the reference values and therefore assumed not to be clinically relevant. Therefore, with the proposed MEMS signal processing pipeline, consisting of outlier rejection, interpolation and filtering, resulting in an average correlation of over *r*=0.95, the MEMS data can be compared for classification purposes (as normal values or not) to reference values collected with a 2D FOG system.

Statistically significant differences were found between the 3D MEMS roll angles in comparison with the 2D FOG values for the most dynamic gait task “get up and go 3 m,” that is, with the greatest range of pitch angular velocity (over 100 deg/s, Table 2). The observed differences between the contingency tables based on 3D MEMS and 2D FOG were due to cross-talk errors of axial rotations and not to noise. When comparing the two contingencies tables (Tables 4 and 5), this happened in less than 1% of all recordings. Specifically, the errors occurred almost exclusively with the get up and go task, which had large jaw and pitch axial rotations (Figure 3). Thus, employing balance tasks with little yaw rotation would avoid this problem. Nonetheless using the third orthogonal, yaw sensing axis of the MEMS opens the possibility of measuring trunk sway during many other clinically relevant balance assessments tasks involving turning (e.g., those of Dite and Temple [16] and Salarian et al. [17]).

Angular velocities measured with the MEMS sensors were obtained by differentiating the processed quaternion output with respect to time. Even if the small differences noted (less than 3% of normal reference values) are not clinically relevant, angular velocities tended to be underestimated by the MEMS. A cause of this difference could be related to mechanical misalignment of the two sensor systems, which is estimated to be around 1 degree [14]. Another cause is likely to be noise and spikes in the MEMS angular velocity data and probably both noise and spikes could be reduced further by modifying the signal processing used here. For example, the bias-corrected gyroscope signal could be sent by the sensor in addition to the quaternion. This, however, would require a modification to the currently used Bluetooth protocol. Additionally, data from multiple MEMS sensor modules could be fused to reduce noise levels. This would require a proper mechanical alignment and time synchronization between the modules. Another alternative would be to improve the filtering of the angular velocity spikes in comparison with the Hampel filter used here.

One of the drawbacks of our study is the limited range of subject ages (19–34 years) we considered. We have compared the accuracy of the two systems in relation to the healthy control reference database of Hegeman et al. [5]. As Hegeman et al. [5] have shown that there are no differences in balance control between the 10 young adults of aged 19–34 years we tested and those aged 35–60, we can argue that our comparison is applicable to patients with the age range 19–60 years, but possibly not for patients less than 19 and persons older than 60 years. Both of the latter groups have sway greater than middle-aged persons [5].

There are three analytical and clinical areas, which should be considered for future studies. As indicated above, the MEMS system tends to underestimate values of pitch and roll velocities. Thus, the cause of this difference should be examined and established if this underestimate is due to signal processing or sensor alignment. If these causes are ruled out, then attention should be placed on examining patient groups with ataxia that are known to have higher velocity trunk sway during gait trials with tandem steps or eyes closed [18]. Underestimates of velocities might prove to be clinically relevant for these patient groups. A third area concerns children younger than 6 years for whom there is no SwayStar reference data [5]. The lighter weight of the MEMS system compared to the FOG system is crucial when considering measurements of this age group.

In this study, the MEMS sensors were attached directly onto the FOG system, which was mounted on a converted motorcycle kidney belt. Therefore, both sensor systems measured the same angular movements of the pelvis and lower back. This ensured that movement of the skin during the tasks had no effect in comparing the measurements in the pitch and roll planes between the sensors systems. MEMS sensors can be mounted with double-sided adhesive tape directly on the skin or with an elasticated belt around the waist. These later methods of mounting can cause distinctive soft tissue artefacts compared to the relatively rigid converted motorcycle belt used for the FOG. Additionally, the significant difference in weight between the two sensor systems can influence the effect of soft tissue movements on the outcome measures. For walking tasks, typical roll and pitch soft tissue errors are of the order of 1–2 degrees [19] and therefore at least equal to 20% of the roll angle amplitudes we measured during gait tasks when the yaw contribution was ignored (Figures 3 and 4; Tables 2 and 3). Given the effect of soft tissue artefacts during dynamic gait balance tasks, and our results indicating that the effect of yaw angle on roll angle estimates was much greater during routine clinical gait tasks compared to stance tasks, we consider it advantageous to concentrate on recording angular velocity measures when using body-mounted sensors to quantify gait balance control pathologies, as roll and pitch velocity measures are not influenced by yaw rotations. In this respect, many current patient classification techniques rely on angle measures for stance and velocity measures for gait [2, 4, 7].

In conclusion, except for tests that involve large yaw movements, there were no significant differences between 2D FOG and 3D MEMS roll and pitch angle measures, although angular velocities were slightly underestimated with the MEMS system. Therefore, 2D MEMS data showed almost perfect agreement with the 2D FOG data. In summary, although for some tasks and some measures statically significant differences were found, further analysis showed that all these differences were within a few percent of the reference values and therefore these differences were assumed not to be clinically relevant. Future studies could consider placing two MEMS sensors side-by-side on a belt, thereby reducing skin artifacts and providing increased accuracy.

## Figures and Tables

**Table 1:** FOG to MEMS comparison results for the task “standing on two legs with eyes open” of all recordings.

Value	PtP Ro A (°)	90 Ro A (°)	PtP Pi A (°)	90 Pi A (°)	PtP Ro V (°/s)	90 Ro V (°/s)	PtP Pi V (°/s)	90 Pi V (°/s)
Mean normal reference	0.493	0.368	1.250	1.004	1.742	0.604	3.311	1.336
FOG mean	0.450	0.322	1.352	1.108	1.553	0.609	2.884	1.404
FOG SD	0.364	0.258	0.481	0.412	0.759	0.261	0.980	0.544
MEMS 2D mean	0.427	0.298	1.337	1.102	1.525	0.595	2.763	1.368
MEMS 2D SD	0.338	0.261	0.484	0.408	0.783	0.266	0.967	0.536
Error between 2D FOG and 2D MEMS relative to mean normal reference	4.66%	6.46%	1.20%	0.63%	1.61%	2.34%	3.67%	2.68%
*p* value (paired *t*-test)	0.242	0.044^*∗*^	0.528	0.739	0.635	0.073	0.079	<0.001^*∗*^
MEMS 3D mean	0.420	0.307	1.299	1.068				
MEMS 3D SD	0.229	0.171	0.338	0.325				
Error between 2D FOG and 3D MEMS relative to mean normal reference	6.16%	4.03%	4.26%	3.97%				
*p* value (paired *t*-test)	0.654	0.769	0.273	0.340				

PtP: peak-to-peak range, 90 : 90% range (95%–5% percentiles); Ro: roll; Pi: pitch; A: angle in degrees; V: angular velocity in degrees/seconds. ^*∗*^Significant difference between the absolute values of FOG and 2D/3D MEMS before any Bonferroni correction. The mean normal reference values are taken from an age-matched group [5].

**Table 2:** FOG to MEMS comparison results for the task “get up and go 3 meters.”

Value	PtP Ro A (°)	90 Ro A (°)	PtP Pi A (°)	90 Pi	PtP Ro V (°/s)	90 Ro V (°/s)	PtP Pi V (°/s)	90 Pi V (°/s)
Mean normal reference	6.451	5.201	45.95	41.90	53.78	29.61	191.7	126.5
FOG mean	5.646	4.347	34.59	31.44	50.61	28.16	139.6	93.18
FOG SD	1.931	1.252	5.858	5.615	23.13	9.960	33.03	27.74
MEMS 2D mean	5.927	4.645	34.57	31.55	48.08	27.78	137.3	92.60
MEMS 2D SD	1.966	1.428	5.876	5.599	21.78	9.768	32.48	26.97
Error between 2D FOG and 2D MEMS relative to mean normal reference	4.35%	5.73%	0.04%	0.26%	4.69%	1.25%	1.18%	0.46%
*p* value (paired *t*-test)	0.077	0.024^*∗*^	0.614	0.167	0.278	0.260	<0.001^*∗*^	0.343
MEMS 3D mean	6.746	5.323	34.54	31.50				
MEMS 3D SD	1.962	1.532	5.997	5.722				
Error between 2D FOG and 3D MEMS relative to mean normal reference	17.0%	18.7%	0.12%	0.15%				
*p* value (paired *t*-test)	0.002^*∗*^	0.002^*∗*^	0.512	0.572				

PtP: peak-to-peak range, 90 : 90% range (95%–5% percentiles); Ro: roll; Pi: pitch; A: angle in degrees; V: angular velocity in degrees/seconds. Note that the differences in 3D are only presented for the angle values; the pitch and roll angular velocities are equal for 2D and 3D. ^*∗*^Significant difference between the absolute values of FOG and 2D/3D MEMS before any Bonferroni correction.

**Table 3:** FOG to MEMS comparison results for the task “walking 8 tandem steps with eyes open.”

Value	PtP Ro A (°)	90 Ro A (°)	PtP Pi A (°)	90 Pi A (°)	PtP Ro V (°/s)	90 Ro V (°/s)	PtP Pi V (°/s)	90 Pi V (°/s)
Mean normal reference	6.324	4.714	6.920	5.160	33.86	18.49	37.92	21.03
FOG mean	5.200	3.718	5.706	4.126	35.93	19.21	31.90	17.14
FOG SD	1.904	1.494	1.328	1.181	12.47	5.055	5.813	3.407
MEMS 2D mean	5.134	3.657	5.717	4.127	34.18	18.76	30.66	16.59
MEMS 2D SD	1.919	1.524	1.336	1.189	11.46	4.963	5.076	3.156
Error between 2D FOG and 2D MEMS relative to mean normal reference	1.04%	1.29%	0.16%	0.01%	5.17%	2.48%	3.28%	2.62%
*p* value (paired *t*-test)	0.202	0.155	0.675	0.984	0.051	<0.001^*∗*^	0.216	0.003^*∗*^
MEMS 3D mean	5.126	3.641	5.957	4.229				
MEMS 3D SD	1.968	1.534	1.132	1.113				
Error between 2D FOG and 3D MEMS relative to mean normal reference	1.17%	1.64%	3.63%	1.98%				
*p* value (paired *t*-test)	0.496	0.447	0.132	0.412				

PtP: peak-to-peak range, 90 : 90% range (95%–5% percentiles); Ro: roll; Pi: pitch; A: angle in degrees; V: angular velocity in degrees/seconds. ^*∗*^Significant difference between the absolute values of FOG and 2D/3D MEMS before any Bonferroni correction.

**Table 4:** Contingency table for agreement on values lying within or outside the range of 95% limit of the reference data.

MEMS 2D	FOG 2D	
	Inside range	Outside range

Inside range	637	1
Outside range	1	33

**Table 5:** Contingency table for agreement on values lying within or outside the range of 95% limit of reference data.

MEMS 3D	FOG 2D	
	Inside range	Outside range

Inside range	636	6
Outside range	2	28

## Data Availability

The data used to support the findings of this study are available from the corresponding author upon request.
